# Adsorption Capacity and Desorption Efficiency of Activated Carbon for Odors from Medical Waste

**DOI:** 10.3390/molecules28020785

**Published:** 2023-01-12

**Authors:** Jung Eun Park, Eun Seo Jo, Gi Bbum Lee, Sang Eun Lee, Bum-Ui Hong

**Affiliations:** Center for Bio Resource, Institute for Advanced Engineering, Yongin-si 17180, Republic of Korea

**Keywords:** activated carbon, odor gas, waste incineration, medical waste, hospital waste

## Abstract

Five types of odor-emitting exhaust gases from medical waste were selected, and their adsorption capacity and desorption efficiency were investigated using activated carbon. The selected gases included polar gases (hydrogen sulfide (H_2_S) and ammonia (NH_3_)) and non-polar gases (acetaldehyde (AA), methyl mercaptan (MM), and trimethylamine (TMA))). Commercial activated carbon with a specific surface area of 2276 m^2^/g was used as the adsorbent. For the removal of odor from medical waste, we investigated: (1) the effective adsorption capacity of a single gas (<1 ppm), (2) the effect of the adsorbed NH_3_ gas concentration and flow rate, and (3) the desorption rate using NH_3_ gas. The values of the effective adsorption capacity of the single gas were in the following order: H_2_S < NH_3_ < AA < MM < TMA, at 0.2, 4.2, 6.3, 6.6, and 35.7 mg/g, respectively. The results indicate that polar gases have a lower effective adsorption capacity than that of non-polar gases, and that the size of the gas molecules and effective adsorption capacity exhibit a proportional relationship. The effective adsorption performance of NH_3_ gas showed an increasing trend with NH_3_ concentration. Therefore, securing optimal conditions for adsorption/desorption is imperative for the highly efficient removal of odor from medical waste.

## 1. Introduction

Medical waste refers to the waste generated in hospitals which can be classified as harmful or harmless [1,2,3,4]. Hazardous medical waste is classified into seven types (damaging, infectious, pathological, pharmaceutical, cytotoxic, chemical, and radioactive); it is generally incinerated. Owing to the COVID-19 pandemic, an increased amount of medical waste was generated worldwide. Additionally, the use of disposable products that require waste treatment also increased [5]. Republic of Korea has the strongest regulation on medical waste followed by Belgium, Germany, Japan, and the United States. Medical waste has gained attention after the Medical Waste Act in 2022, which provided a revision stating that medical waste can be discarded as workplace waste. However, research is required.

Current methods for the treatment of medical waste are limited by legal restrictions in each country, and the on-site treatment rate is generally low. Non-incineration methods for on-site treatment can be divided into thermal processing (autoclave), microwave technology, chemical technology, and dried sterilization. Among them, thermal processing is one of the preferred methods to achieve sterilization before transportation and is widely used for instrument sterilization [6]. After thermal processing and microwaving, sterilized waste is incinerated. However, incineration requires high temperatures and leads to the production of various environmental pollutants, such as tetrachlorodibenzo-p-dioxide (TCDD), persistent organic pollutants, and total volatile organic compounds (TVOCs) [7,8].

In previous research, the medical waste was treated with steam, microwave, and chemical disinfection methods [8,9]. Among them, it is recommended to treat medical waste in parallel with microwave and thermal processes to increase the removal efficiency of pollution from medical waste. After treatment, the VOC concentration in the exhaust gas at the outlet of the steam treatment was substantially higher than that in the microwave and chemical treatment. In contrast, for chemical treatment, the VOC concentration in workshops was higher than that for steam and microwave treatments. Methyl-sulfhydryl, dimethyl sulfide, methyl disulfide, and hydrogen sulfide concentrations in actual medical waste have been detected at 0.037, 0.046, 0.904, and 0.204 mg/m^3^, respectively. After biological deodorization by steam treatment, the average removal rates of these four odor gases were 93, 92.6, 97.6, and 92.6%, without exact concentrations [10]. The treatment of medical waste must destroy pathogenic bacteria to eliminate their infection ability. Among the three abovementioned treatment methods, steam processing presents the best sterilization effect. Previous studies have analyzed the properties of exhaust gases emitted after microwave and steam treatments. However, there is a lack of information on the removal efficiency and accurate final concentrations compared to the input. Previous studies have recommended the treatment of medical waste by both microwave and thermal process to increase sterilization efficiency [11]. However, research on the treatment of exhaust gas generated by medical waste and sterilization processes is insufficient.

Exhaust gases from medical waste are challenging to treat owing to their varied properties and concentrations [12,13,14]. Generally, exhaust gases can be treated by absorption, adsorption, combustion, and biochemical treatments. Treatments based on absorption and biochemical methods do not exhibit high removal efficiency, and adsorption treatments require a high system volume, high flow rate, and treat only low concentrations of exhaust gas. To apply an adsorption treatment, the adsorbent should exhibit a high adsorption capacity toward many exhaust gases.

Adsorption experiments have been conducted using zeolite, silicate, activated carbon, and activated carbon fibers [15]. Activated carbon has micropores, mesopores, and macropores with nonpolar and hydrophobic characteristics. Because of these characteristics, activated carbon is widely used in the study of VOC’s gas adsorption of BTEX series [16,17]. Generally, activated carbon has a high adsorption performance toward non-polar gases such as VOCs [18], whereas polar chemicals (NH_3_ and H_2_S) with high adsorption potential are generally poorly adsorbed [19]. Recently, activated carbon has been considered suitable for the adsorption of amine gases because a well-developed pore structure and large specific surface area are preferred for the functional (acidic or basic) groups on the adsorbent surface. In contrast, a few studies showed that adsorption capacity is related to more acidic and less stable oxygen surface groups [20,21]. However, the adsorption process of exhaust gases from medical waste has not been fully investigated due to a disease outbreak and legal constraints [2,4]. In this study, we investigated the removal efficiency of activated carbon for major exhaust gases from medical waste. The objectives of this study were: (1) to compare the effective adsorption capacity of activated carbon for different adsorbate gases, such as the aldehyde group (acetaldehyde), amine group (ammonia and trimethylamine), and sulfide group (hydrogen sulfide and methyl mercaptan), (2) to evaluate adsorption factors such as space velocity (SV) and initial concentration, and (3) to evaluate the respective desorption properties.

## 2. Results

### 2.1. Properties of Activated Carbon

Based on the proximate composition analysis, the activated carbon was composed of 1.1% moisture, 4.0 wt% ash, 17.3 wt% volatile matter, and 77.5 wt% fixed carbon, as shown in Table 1. The fixed carbon content is inversely proportional to moisture, ash, and volatile contents [22]. Additionally, 82.0% carbon, 0.8% hydrogen, and 10.5% oxygen content were obtained from the dried material. The carbon content was similar to that of previous research on coal-based activated carbon [23].

**Figure 1:**
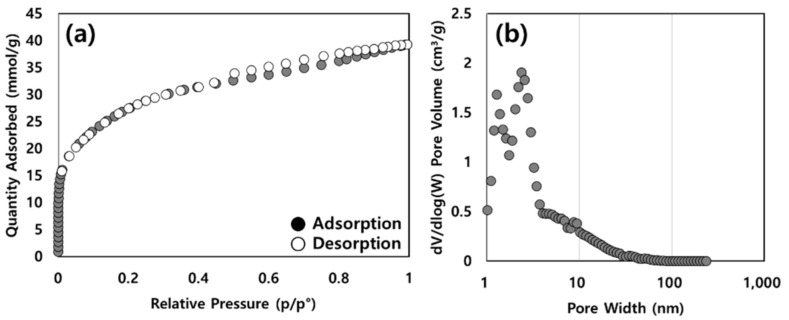
(**a**) Nitrogen adsorption–desorption isotherms and (**b**) pore size distribution of activated carbon.

The shape of the nitrogen adsorption–desorption isotherms is illustrated in Figure 1, which shows a type IV isotherm with a mixture of microporous and mesoporous materials. Based on the results, the BET surface area was 2276 m^2^/g, the pore size was 0.89 cm^3^/g, and the pore diameter was 2.95 nm.

**Figure 2:**
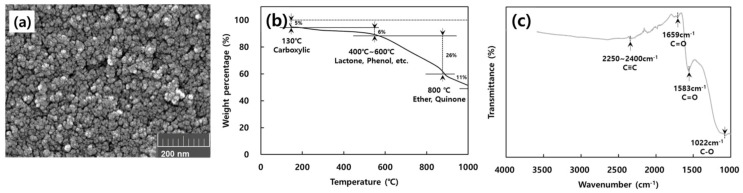
(**a**) Scanning electron microscopy (SEM) image, (**b**) TGA weight percentage curve, and (**c**) spectrogram of activated carbon.

The surface morphology of activated carbon could be observed on the view of microcosm through scanning electron microscopy (SEM) analysis. The SEM image in Figure 2a shows that the activated carbon consisted of small lumps with many pores and was well-developed on the surface. The highly developed mesopore in the SEM image has a relatively higher BET surface and pore size distribution. Thermogravimetric analysis (TGA) and Fourier transform infrared spectroscopy (FT-IR) were used to observe the variation of weight loss and active functional groups on the surface of the adsorbent. TGA analysis of activated carbon was conducted to investigate its thermal stability, and the results are shown in Figure 2b. The TGA curve shows that the weight of activated carbon started to drop slowly. During the weight loss, until 130 °C it decomposed into carboxyl groups, and during the gradual weight loss until 600 °C it decomposed into lactone and phenol groups. At 800 °C, the weight loss occurring was due to the decomposition of ether and quinone groups [24]. After around 1000 °C, the weight of activated carbon remained at about 51.6% of the initial weight. The FT-IR spectrums for activated carbon are shown in Figure 2c. The functional groups of activated carbon showed three main peaks identified at 1022 cm^−1^, 1583 cm^−1^, and 1659 cm^−1^, which were involved in the stretching of the C-O and C=O bonds in the carboxyl groups, respectively. The presence of a carbon–carbon triple bond in disubstituted alkynes can be inferred from the bands in the range of 2250–2400 cm^−1^. However, the band was not found in the 2500~4000 cm^−1^ range for hydroxy groups (O-H), asymmetrical or symmetrical (C-H) stretching [25].

### 2.2. Adsorption Properties of Activated Carbon toward Exhaust Gases

#### 2.2.1. Effective Adsorption Capacity for Different Exhaust Gases

To evaluate the adsorption properties of activated carbon, its adsorption capacity should be investigated. The odor threshold and irritation level of exhaust gases is in the parts per billion level [26]. The total adsorption capacity of adsorbent is difficult to use as a design factor for equipment that removes exhaust gases from medical waste. Therefore, we calculated the effective adsorption capacity of less than 1 ppm based on the breakthrough point [27].

**Figure 3:**
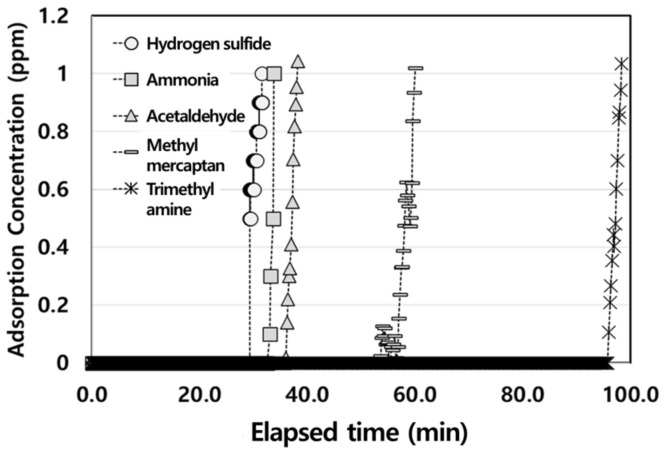
Breakthrough curve of exhaust gases on activated carbon. Total flow rate is 1 L/min and initial feed concentration is 100 ppm. The feeding gases were hydrogen sulfide (H_2_S), ammonia (NH_3_), acetaldehyde (AA), methyl mercaptan (MM), and trimethylamine (TMA).

Breakthrough column experiments were performed in a stainless steel 304 tubular reactor using a total flow of 1 L/min containing hydrogen sulfide (H_2_S), ammonia (NH_3_), acetaldehyde (AA), methyl mercaptan (MM), and trimethylamine (TMA), as shown in Figure 3. The breakthrough curve appeared in the order of H_2_S, NH_3_, AA, MM, and TMA. Compared to the exhaust gases, TMA presents a better dynamic adsorption performance owing to the longer breakthrough time and corresponding larger effective adsorption capacity (<1 ppm). The effective adsorption capacities (mg/g) of the different samples are listed in Table 2.

The results suggest that the effective adsorption capacity (<1 ppm) of activated carbon toward exhaust gases is proportional to the size of the pollutant molecules. If the molecular size of target pollutants is close to the pore size, then it can be easily adsorbed [26]. The optimal ratio of the pore size to the pollutant molecular size that is required to obtain an excellent adsorption performance is between 1.7 and 3.0 [28]. Therefore, the pollutant molecular size and effective adsorption capacity exhibit a proportional relationship. Furthermore, the results of this study cannot be compared with previous research because the activated carbon used for H_2_S adsorption is generally investigated by total adsorption capacity (~100%) [29]. A novel adsorbent-Cu/activated carbon was evaluated for H_2_S, NH_3_, and TMA adsorption, which exhibited total adsorption capacities of 164.54, 190.68, and 323.56 mg/g [30], respectively, which are in the same order as the results of our research. The total adsorption capacity is different in each study because the adsorption mechanisms vary based on adsorption conditions. Guo et al. developed an intra-particle Knudsen diffusion model based on the Freundlich isotherm to predict the adsorbed amount of H_2_S [29]. Another study investigated the correlation between initial H_2_S concentration and adsorption capacity, and the H_2_S adsorption followed the Langmuir isotherm and pseudo-first kinetics models [30,31]. In addition, the Langmuir isotherm model was the best fit in describing the adsorption of AA on NaOH/AC filter and TMA on Cu/AC [32,33]. Therefore, we concluded that it would be more accurate to evaluate the effective adsorption capacity at a high adsorption capacity (<1 ppm).

#### 2.2.2. Effect of SV on NH_3_ Gas

The effective adsorption capacity of activated carbon for ammonia is influenced by changes in the gas-phase SV. The flow rate was increased from 0.1 to 10 L/min. The results indicate that the contact time was sufficient for gaseous ammonia to flow through the interpores and intrapores, reach the stagnant area, and adhere to the adsorbent bed [34].

**Figure 4:**
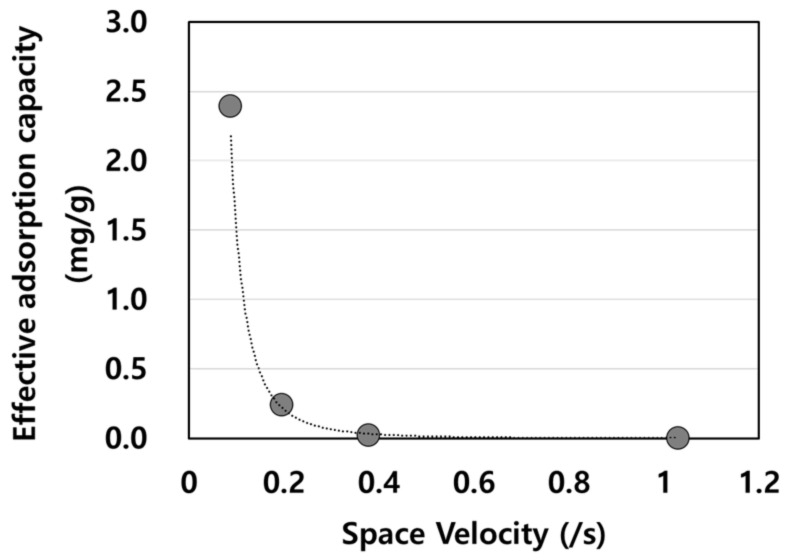
Effective adsorption capacity of NH_3_ based on space velocity.

Figure 4 shows that the effective adsorption capacity is inversely proportional to SV. The higher flow rate contrasted with the fact that the majority of ammonia can escape from the sorbent surface sites because of its high input load at the inlet [35,36]. This suggested that the kinetics of ammonia captured on the adsorbent should be controlled.

#### 2.2.3. Effect of NH_3_ Gas Concentration

**Figure 5:**
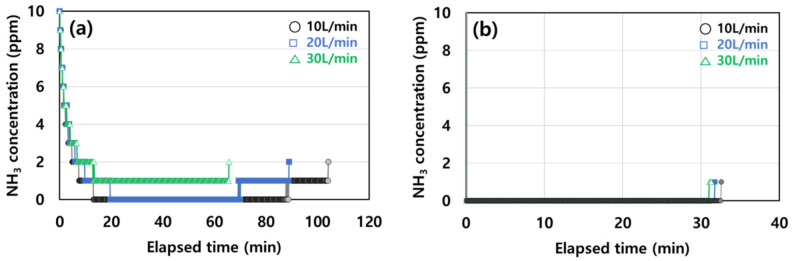
Effect of flow rate under different NH_3_ feed concentrations at (**a**) 40 ppm and (**b**) 1000 ppm on the adsorption performance of activated carbon.

In the case of ammonia, the effect of the initial concentration and flow rate on the adsorption efficiency was studied. At a feeding concentration of 40 ppm, the adsorption rate presents a gentle slope, and the effective adsorption capacity changes based on the flow rate in Figure 5a. The flow rate increased as the effective adsorption capacity decreased even if the baseline of adsorption did not reach 0 ppm, while it reached 1 ppm at 30 L/min. As the flow rate was increased to 10, 20, and 30 L/min, the effective adsorption capacity decreased to 4.9, 4.1, and 2.9 mg/g, respectively.

Figure 5b shows that the adsorption time and effective adsorption capacity decreased with an increasing flow rate at a higher input concentration (1000 ppm). The flow rate increased to 10, 20, and 30 L/min as the effective adsorption capacity decreased to 11.0, 10.6, and 9.8 mg/g, respectively. Comparing Figure 5a and 5b, we concluded that a higher initial input of ammonia gas led to a higher adsorption capacity [35].

### 2.3. Desorption Properties of NH_3_

#### 2.3.1. Desorption Pattern for Different Adsorbed NH_3_ Concentrations

**Figure 6:**
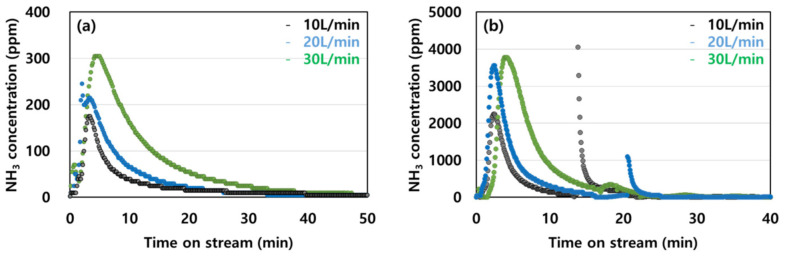
Effects of desorption flow rate on the adsorption of NH_3_ at concentrations of (**a**) 40 ppm and (**b**) 1000 ppm at 100 °C on activated carbon.

The desorption of ammonia was analyzed for different concentrations (40 ppm and 1000 ppm) and flow rates (10, 20, and 30 L/min), and the results are shown in Figure 6. The maximum desorbed concentration differs based on the adsorption concentrations. However, the results indicate that ammonia was desorbed after being concentrated by up to ten times. The desorption rate according to the desorption flow rate was examined using the adsorbent at a concentration of 40 ppm. Approximately 60% of the adsorbed ammonia was desorbed after approximately 1.8 h at a flow rate of 10 L/min, and approximately 79% was desorbed after 1.5 h at 20 L/min. At the highest desorption flow rate of 30 L/min, approximately 81% ammonia was desorbed after 1.4 h. The results are indicative of a tailing phenomenon, in which desorption occurs at a low flow rate over an extended period of time because the energy required for desorption is inadequate. At a concentration of 1000 ppm, the desorption rate increased and the desorption time decreased as the desorption flow rate increased. Owing to the initial feed concentration of 1000 ppm, the desorption rates of 50, 79, and 80% required adsorption times of 2.4, 2.3, and 1.4 h, respectively.

The results show that the desorption amount increased with the flow rate regardless of the adsorption concentration because the desorbed amount was proportional to the desorption energy.

#### 2.3.2. Desorption Flow Rate and Temperature

**Figure 7:**
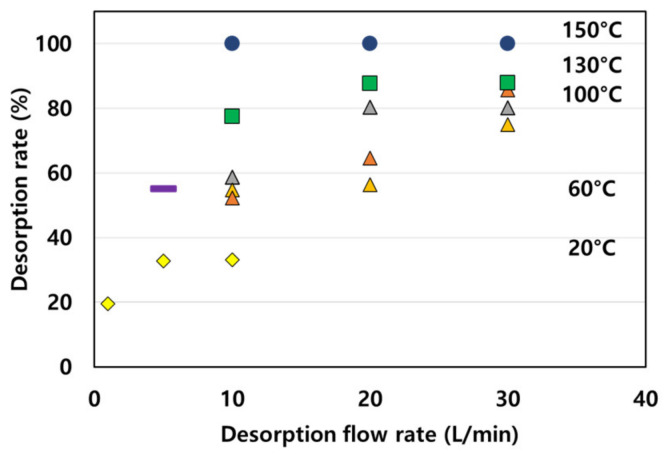
Desorption rate based on desorption flow rate and desorption temperature (● 150 °C, ■ 130 °C, ▲ 100 °C, ─ 60 °C, and ◆ 20 °C).

The results indicate that desorption was proportional to the desorption flow rate. However, to identify optimal desorption conditions, 1000 ppm of ammonia was efficiently adsorbed at 1 L/min, and the desorption rate was analyzed for different desorption temperatures and desorption flow rates. The results are shown in Figure 7.

The experiments performed under different desorption conditions using ammonia gas led to a low desorption rate of ≤60% at a desorption temperature of ≤60 °C. At a desorption temperature of ≥100 °C, the desorption rate increased with the flow rate. At 150 °C, the desorption flow rate decreased as the desorption temperature increased, and 100% desorption occurred regardless of flow rate. Therefore, desorption energy was affected by temperature and flow rate, and the desorption rate was not affected by the desorption flow rate at 150 °C. Thus, we concluded that the desorption energy is adequate at 150 °C.

## 3. Materials and Methods

### 3.1. Materials

#### 3.1.1. Activated Carbon Adsorbent

To investigate the removal efficiency of exhaust gases from medical waste, the activated carbon adsorbent was purchased as the granular type, with a size of 3.0–4.0 mm (CS Company, Seongnam, Republic of Korea). The material was dried at 110 °C for 24 h before the experiment. The proximate and ultimate analyses of activated carbon are shown in Table 3. The adsorbent was analyzed by proximate analysis, elemental analysis (EA), and N_2_ adsorption/desorption properties. For the proximate analysis, the dried adsorbent carbon was placed in a furnace (DF-4S, Daeheung Science, Inchen, Republic of Korea) and heated at 950 °C for 7 min and then at 750 °C for 10 h. The sample products, including ash, volatile, and fixed carbon contents, were measured as percentages of the total weight [37]. EA was conducted for 12 min at 900 °C using an elemental analyzer (628 Series, LECO, St. Joseph, MI, USA) to determine the carbon, hydrogen, oxygen, nitrogen, and sulfur concentrations [38].

The surface area and pore structure characteristics of the activated carbon were determined by nitrogen adsorption at −196 °C by a surface area analyzer ASAP 2020 (Micromeritics, Norcross, GA, USA). The surface area (S_BET_) of the prepared activated carbon was estimated by the Brunauer–Emmett–Teller (BET) method. The pore size distribution of the activated carbon was determined using the Barrett–Joyner–Halenda (BJH) desorption model, and the mean pore diameter (D_p_) was calculated from D_p_ = 4Vt/S_BET_ [39].

To investigate surface morphologies of activated carbon, field-emission scanning electron microscopy (S-4300, Hitachi, Tokyo, Japan) was used. In addition, the thermal decomposition characteristics of the activated carbon surface oxygen functional groups (OFGs) were confirmed through a thermogravimetric analyzer (TGA4000, Perkin Elmer, Waltham, MA, USA). The presence of the activated carbon surface functional groups was confirmed via Fourier transform infrared spectroscopy with the ATR method (Nicolet iS50, Thermo Fisher Scientific, Waltham, MA, USA).

#### 3.1.2. Exhaust Gases Detected from Medical Waste

The effective adsorption capacity of activated carbon toward five types of gases, exhausted from medical waste, was evaluated. The concentration of the exhaust gases (JC gas, Anseong, Republic of Korea) was balanced using N_2_, including 1% hydrogen sulfide (H_2_S), 1% ammonia (NH_3_), 1% acetaldehyde (AA), 1% methyl mercaptan (MM), and 100 ppm trimethylamine (TMA). The properties of the exhaust gases from medical waste are shown in Table 3.

**Table 3:** Textural properties of exhaust gases from medical waste.

Gas	Polar		Nonpolar		
	Hydrogen Sulfide	Ammonia	Acetaldehyde	Methyl Mercaptan	Trimethylamine
	H_2_S	NH_3_	CH_3_CHO	CH_3_SH	C_3_H_9_N
M.W. (g/mol)	34.08	17.031	44.05	48.11	59.11
Molecular Size (Å)	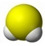	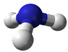	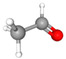	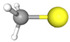	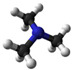
	1.89	3.45	3.82	4.37	6.57

### 3.2. Adsorption/Desorption Methods

Prior to the adsorption tests, the activated carbon was dried and placed in a reactor. The adsorbent weight was 27.50 ± 0.05 g (weight ± deviation). The adsorption filters were placed in a fixed-bed reactor inside a stainless steel 304 tubular adsorption chamber. For the adsorption experiment, each standard gas was continuously injected into the system in an enclosed adsorption chamber. The standard gas achieved effective desorption after effective adsorption. The monitoring equipment MultiRAE Pro (SungHwa electron, Busan, Republic of Korea) was used to continuously monitor the concentrations of H_2_S and NH_3_, while Polaris FID (Pollution, Budrio, Italy) was used for AA, MM, and TMA. The measuring instrument performed detections at 1 s intervals, and effective adsorption capacity was based on values under 1 ppm. After effective adsorption, desorption was performed at different temperatures from 20 to 150 °C.

## 4. Conclusions

In this study, the effective adsorption capacity of an adsorbent was evaluated using five types of pollution exhaust gases generated from medical waste, including hydrogen sulfide, ammonia, acetaldehyde, methyl mercaptan, and trimethylamine. To compare the effective adsorption capacity of exhaust gases, commercial activated carbon was used as the adsorbent. The effective adsorption capacities of trimethylamine, methyl mercaptan, acetaldehyde, ammonia, and hydrogen sulfide were 35.7 mg/g, 6.6 mg/g, 6.3 mg/g, 4.2 mg/g and 0.2 mg/g, respectively. The effective adsorption capacity was proportional to the molecular size, and the polar gases (hydrogen sulfide and ammonia) led to lower effective adsorption capacities than the non-polar gases (acetaldehyde, methyl mercaptan, and trimethylamine). Experiments were performed under each adsorption condition for ammonia, a polar gas. The effective adsorption capacity of activated carbon was inversely proportional to the SV, and effective adsorption capacity increased with the supply concentration. In contrast, the effective adsorption capacity decreased as the flow rate increased. Moreover, the supply of a high concentration at a low flow rate during the adsorption process led to a high effective adsorption capacity. The desorption rate was proportional to the desorption flow rate and desorption temperature. At 150 °C, 100% of the adsorbed ammonia was desorbed. In conclusion, experiments were performed under different adsorption/desorption conditions to remove odors from medical waste. The results indicate that optimal adsorption/desorption conditions should be ensured based on the properties and concentrations of the generated odors.

## Figures and Tables

**Table 1:** Proximate and ultimate analyses of activated carbon.

Proximate Contents (%)		Ultimate Contents (%)	
Moisture	1.1	Carbon	82.0
Fixed Carbon	77.5	Hydrogen	0.8
Volatiles	17.3	Nitrogen	0.1
Ash	4.0	Oxygen	10.5
Sum	100.0	Sulfur	0.0

**Table 2:** Effective adsorption capacity (<1 ppm) of activated carbon toward five different gases.

Gas	H_2_S	NH_3_	AA	MM	TMA
Effective adsorption capacity (mg/g)	0.2	4.2	6.3	6.6	35.7
